# ECG non-specific ST-T and QTc abnormalities in patients with systemic lupus erythematosus compared with rheumatoid arthritis

**DOI:** 10.1136/lupus-2016-000168

**Published:** 2016-12-16

**Authors:** Laura Geraldino-Pardilla, Yevgeniya Gartshteyn, Paloma Piña, Marina Cerrone, Jon T Giles, Afshin Zartoshti, Joan M Bathon, Anca D Askanase

**Affiliations:** 1 Columbia University, College of Physicians & Surgeons, New York, New York, USA; 2 Northwestern University, Chicago, Illinois, USA; 3 New York University (NYU) School of Medicine, New York, New York, USA

**Keywords:** Cardiovascular Disease, Systemic Lupus Erythematosus, Autoimmune Diseases

## Abstract

**Objectives:**

Cardiovascular disease (CVD) is a leading cause of death in systemic lupus erythematosus (SLE) and in rheumatoid arthritis (RA). Although only explored in one study, ECG non-specific ST-T abnormalities, in addition to corrected QT-interval (QTc) prolongation, were recently reported in an SLE inception cohort. Importantly, these ECG abnormalities are known predictors of CVD mortality in the general population, yet their prevalence in patients with established SLE has not been evaluated.

**Methods:**

We cross-sectionally investigated the presence of non-specific ST-T and QTc abnormalities in 50 patients with SLE, predominantly Hispanic and black, without CVD or SLE-related cardiac involvement and compared them with 139 patients with RA without CVD. Demographics, disease-specific characteristics and CVD risk factors were ascertained and adjusted for.

**Results:**

Patients with SLE (mean age 36±13 years, 92% women, 6 years median disease duration, 96% Hispanics and blacks) had a 3.3-fold higher adjusted prevalence of non-specific ST-T abnormalities (56% vs 17%; p <0.0001) compared with RA, despite the older age and higher percentage of men in the RA group. The QTc was 26 ms longer in SLE compared with RA (p=0.002) in the setting of a higher percentage of women, blacks, Hispanics and higher C reactive protein levels in the SLE group.

**Conclusions:**

This study demonstrates a high prevalence of ECG abnormalities in predominantly Hispanic and black patients with SLE. Longitudinal evaluation of the progression to potentially life-threatening arrhythmias and/or cardiovascular events is warranted.

## Introduction

Cardiovascular disease (CVD) is a leading cause of death in patients with systemic lupus erythematosus (SLE).^1^ ^2^ Despite improvements in overall survival in the past decades, mortality due to CVD in SLE remains unchanged.^3^ Compared with age-matched controls, studies in SLE have reported a twofold to threefold increased risk of cardiovascular (CV) mortality and congestive heart failure, and a twofold to 10-fold increased risk of myocardial infarction (MI); with a relative risk as high as 52.4 (95% CI 21.6 to 98.5) in those 35–44 years of age over an average follow-up of 6.7 years.^4^ In addition to conventional CV risk factors, SLE itself is an independent risk factor for CVD.^5^ This makes traditional CV risk stratification scores developed for the general population underperform in identifying patients with SLE at high risk for CVD.^6^ Consequently, there is a growing interest in improving CV risk stratification in SLE; ECG, an inexpensive yet reproducible diagnostic tool, could serve this purpose by potentially detecting SLE-associated cardiac involvement.

Although only explored in one recent study, ECG non-specific ST-T abnormalities were reported in 31% of patients with SLE from an inception cohort; additionally, a prolonged corrected QT-interval (QTc) was noted in 15% of patients.^7^ However, Bourré-Tessier *et al* lacked a comparison group and the patients consisted of predominantly whites and Asians with mild disease activity. Hence, the prevalence of ECG non-specific ST-T and QTc abnormalities in patients with established SLE and multiethnic backgrounds (with blacks and Hispanics known to have more severe lupus), and/or at more advanced stages of clinical disease, remains unclear. Importantly, these ECG abnormalities are known predictors of cardiovascular mortality in the general population,^8^ ^9^ yet their impact in CV risk in SLE is not known.

In rheumatoid arthritis (RA), an autoimmune disease with similarly increased CVD risk,^10^ ECG abnormalities such as QTc prolongation have been reported in association with a doubling risk for all-cause mortality.^11^ These characteristics make RA a good control group for patients with SLE, despite the differences in age/sex between the groups.

The present study focused on the prevalence of non-specific ST-T and QTc abnormalities in a cohort of patients with SLE of predominantly Hispanic and black ethnicities, without clinical CVD. We compared the SLE group with patients with RA without clinical CVD and hypothesised that the prevalence of ECG abnormalities of interest would be higher in the patients with SLE relative to RA, as well as to the previously studied SLE cohort described by Bourré-Tessier *et al*.^7^

## Materials and methods

### Study population

#### Patients with SLE

Fifty patients with SLE randomly recruited from the Columbia University Lupus Cohort between January and June 2015 were evaluated at a single visit. All patients were 18 years of age or older and met 1997 American College of Rheumatology (ACR) classification criteria.^12^ Exclusion criteria included known CVD at baseline (defined as self-reported physician-diagnosed MI, heart failure, coronary artery revascularisation, angioplasty, peripheral vascular disease, implanted pacemaker or defibrillator devices and current atrial fibrillation) or a diagnosis of current pericarditis or myocarditis at the time of enrolment; patients with the following ECG abnormalities were also excluded: major ST-T changes, bundle branch block or paced rhythm.

#### Patients with RA

One hundred and thirty-nine patients who underwent 12-lead ECG as part of their enrolment in the Evaluation of Subclinical Cardiovascular Disease and Predictors of Events in Rheumatoid Arthritis (ESCAPE-RA) study were used as controls. ESCAPE-RA is a prospective cohort study of patients with RA established to investigate subclinical CVD as previously described in detail.^13^ All participants were aged 45–84 years, met the ACR 1987 classification criteria for RA and were diagnosed with RA for ≥6 months.^14^ Patients were recruited from the Johns Hopkins Arthritis Center and referrals from community rheumatologists. Similar exclusion criteria as for the SLE group were used for the patients with RA.

### Outcome measures

#### ECG abnormalities

The 12-lead ECGs (25 mm/s paper speed and 10 mm/mV amplitude) were interpreted by two board-certified cardiologists specialised in electrophysiology, blinded to the clinical information. (*A) Non-specific ST-T abnormalities* were defined according to the conventionally used Minnesota codes 4-3 (downwards sloping ST segment with J depression <0.5 mm in leads I, II, augmented vector left electrocardiographic lead (aVL), V2-V6), 4-4 (upwards sloping ST segment from a J point depressed ≥1 mm in I, II, aVL, V1-V6), 5-3 (negative or flat T-wave of <1 mm amplitude in I, II, aVL, V3-V6) and 5-4 (positive but <1/20 of the R amplitude T-wave in I, II, aVL, V3-V6).^15^ *(B) The QT-interval* was calculated and adjusted for the heart rate using Bazett's formula (QTc=QT/√RR).^16^ There is no consensus on defining a normal QTc range, with proposed upper limits of normal extending from 430 to 470ms.^17^ ^18^ We therefore performed several analyses at different cut-off points for QTc, defined as QTc ≥420 ms, ≥440 ms, ≥460 ms and ≥480 ms.

### Clinical covariates

Demographics and smoking history were self-reported and collected by questionnaires. Hypertension was defined as a systolic blood pressure (BP) of ≥140 mm Hg, diastolic BP of ≥90 mm Hg at the time of the evaluation or antihypertensive medication use. Diabetes was defined as a fasting serum glucose of ≥126 mg/dL, glycated haemoglobin (HbA1c) greater than 6.4% or antidiabetic medication use. All medications were documented from patient interview, medical records and containers provided by the study participants. QT-modifying medications were defined as any medication from the following categories: muscle relaxants, antidepressants, antipsychotics, antiemetics and antimicrobials (antivirals/macrolides/fluoroquinolones). SLE and RA disease durations were designated as the duration in years from the date of physician diagnosis. SLE disease activity was calculated using the Systemic Lupus Erythematosus Disease Activity Index 2000 (SLEDAI-2K).^19^ RA activity was calculated using the Disease Activity Score in 28 joints (DAS28) with C reactive protein (CRP) level.^20^

### Laboratory covariates

*SLE-pertinent laboratories* including anti-anti-Sjogren's syndrome-related antigen A (SSA)/Ro, anti-anti-Sjogren's syndrome-related antigen B (SSB)/La, anti-ds-DNA, anti-Smith, anti-(ribonucleoprotein antibody) RNP, antiphospholipid antibodies, CRP and complement levels (C3, C4) were performed at the clinical laboratory at New York Presbyterian Hospital. *RA-pertinent laboratories* including high-sensitivity CRP, interleukin 6 (IL-6), rheumatoid factor (RF) and anticitrullinated cyclic peptide (anti-CCP) antibodies were measured as previously described.^13^

### Statistical analysis

Summary statistics for outcomes and predictor variables were calculated, with comparisons made using Student's t-test and Wilcoxon rank-sum test for normally and non-normally distributed continuous variables, respectively. Counts and percentages were calculated for categorical variables and compared using χ^2^ or Fisher's exact test as appropriate. The association of non-specific ST-T abnormalities with different cut-offs of QTc duration (≥420 ms, ≥440 ms, ≥460 ms, ≥480 ms) with SLE versus RA as the independent variable was explored using logistic regression and adjusted for confounders. Linear regression was used to test the association between QTc length, used as a continuous variable, and SLE versus RA status, adjusting for confounders. To isolate the association between SLE versus RA and the tested ECG abnormalities, confounders were defined as those variables associated with both the outcome (non-specific ST-T and QTc abnormalities) and the independent variables (SLE vs RA status). Additionally in each group (RA and SLE), we separately tested the association between independent variables and the presence of non-specific ST-T and QTc lengths, using χ^2^ and Fisher's exact tests or Student's t-test and Wilcoxon rank-sum test, as well as univariate linear regression as appropriate. Subsequently, multivariate models were constructed using logistic regression and linear regression. Statistical calculations were performed using SAS V.9.4. A two-tailed α of 0.05 was defined as the level of statistical significance for all tests.

## Results

### Patient characteristics

Patient characteristics are shown in table 1. The patients with SLE had a median SLEDAI-2K score of 6 units, 56% had positive anti-SSA/Ro antibodies, 40% had lupus nephritis and none had end-stage renal disease. In the patients with RA, the average DAS28-CRP was 3.6 units, 68% were anti-CCP antibody positive, 61% were RF positive and 48% were on biologics. Patients with SLE were younger; more likely to be woman; use glucocorticoids, antimalarials and QT-modifying medications and currently smoke. In the RA group, only 1% were black or Hispanic and 61% were women; patients with RA were older, had longer disease duration and were more likely to use aspirin and have moderate to severe disease activity based on the DAS28-CRP score, although they had lower CRP levels, on average, compared with the patients with SLE. No difference was seen in the prevalence of hypertension (HTN), diabetes or the use of statins between the SLE and RA groups.

**Table 1:** Patient characteristics

	SLE (n=50)	RA (n=139)	p Value
Age, years	36±13	59±8	**<0.0001**
Female, n (%)	46 (92%)	85 (61%)	**<0.0001**
Race/ethnicity			
White, n (%)	2 (4%)	121 (87%)	**<0.0001**
Hispanic, n (%)	37 (74%)	1 (1%)	**<0.0001**
Black, n (%)	11 (22%)	1 (1%)	**<0.0001**
Disease duration, years	6 (2–10)	8 (4–16)	**0.0005**
SLEDAI-2K	6 (2–12)	–	–
Lupus nephritis, n (%)	22 (44%)	–	–
APS, n (%)	3 (6%)	–	–
Moderate–severe disease activity*, n (%)	21 (42%)	86 (62%)	**0.02**
DAS28	–	3.6±1	–
CRP, mg/L	4.7(1.0–31.5)†	2.1 (1–5.9)	**0.03**
IL-6, pg/mL	–	3.6 (1.6–7.7)	–
Antimalarials, n (%)	32 (64%)	23 (17%)	**<0.0001**
Mycophenolate mofetil, n (%)	21 (42%)	–	–
Azathioprine, n (%)	5 (10%)	–	–
Non-biologic DMARD, n (%)	–	117 (84%)	–
Biologic DMARD, n (%)	–	66 (48%)	–
Current glucocorticoid use, n (%)	27 (54%)	50 (36%)	**0.03**
ds-DNA antibody, n (%)	41 (82%)	–	–
SSA antibody, n (%)	28 (56%)	–	–
SSB antibody, n (%)	16 (32%)	–	–
Sm antibody, n (%)	24 (48%)	–	–
RNP antibody, n (%)	29 (58%)	–	–
RF>40 units, n (%)	–	85 (61%)	–
Anti-CCP>60 units, n (%)	–	94 (68%)	–
Hypertension, n (%)	18 (36%)	56 (40%)	0.59
Diabetes, n (%)	3 (6%)	10 (7%)	0.77
Current smoking, n (%)	12 (24%)	13 (9%)	**0.008**
Aspirin use, n (%)	8 (16%)	44 (32%)	**0.03**
Statin use, n (%)	5 (10%)	23 (16%)	0.26
QT-modifying medication use, n (%)	16 (32%)	24 (17%)	**0.03**
Muscle relaxant, n (%)	1 (2%)	8 (6%)	0.62
Antipsychotics, n (%)	3 (6%)	0	0.98
Antidepressants, n (%)	10 (20%)	23 (17%)	0.67
Antimicrobials‡, n (%)	1 (2%)	2 (10%)	0.79
Antiemetics, n (%)	3 (6%)	3 (2%)	0.08

Characteristics are expressed as n (%), as the mean±SD or as the median (IQR).

*Moderate–severe disease activity is defined as SLEDAI-2K >6 or DAS-28 CRP >3.2.

†n=40.

‡Antimicrobials include macrolides, fluoroquinolones and HIV retrovirals.

Anti-CCP, anticitrullinated cyclic peptide; APS, antiphospholipid antibody syndrome; CRP, C reactive protein; DAS28, Disease Activity Score in 28 joints; DMARD, disease-modifying antirheumatic drug; IL-6, interleukin 6; RA, rheumatoid arthritis; RF, rheumatoid factor; SLE, systemic lupus erythematosus; SLEDAI-2K, Systemic Lupus Erythematosus Disease Activity Index 2000.

Statistically significant p values <0.05 are depicted in bold.

### ECG abnormalities per study group

**Figure 1:**
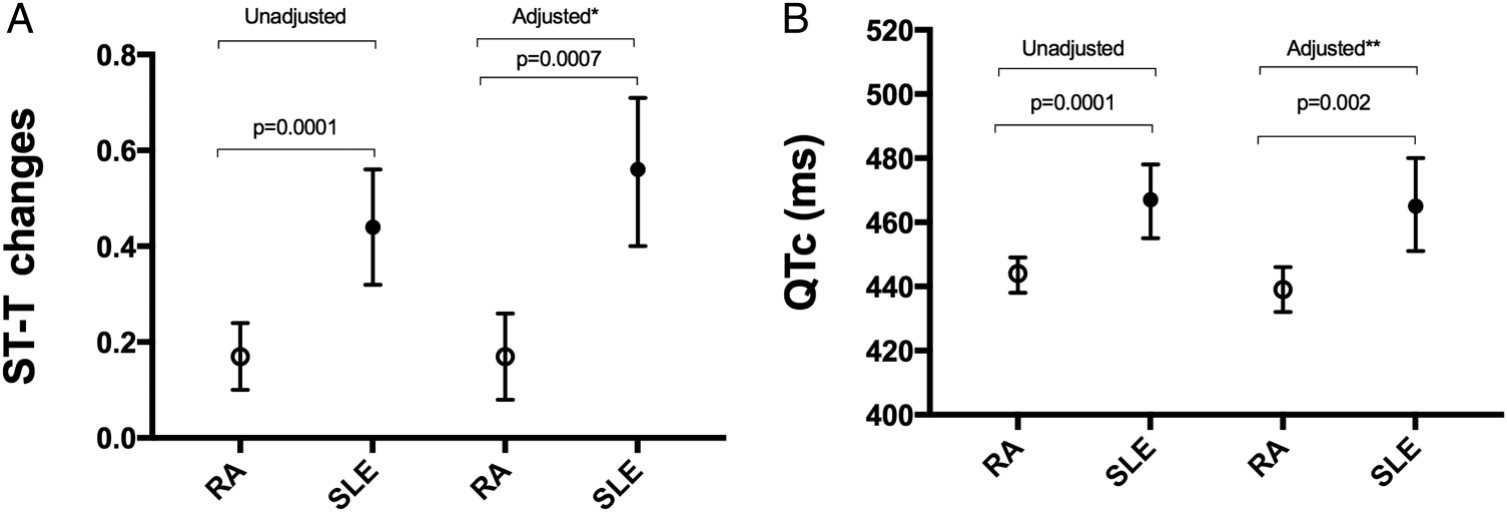
Prevalence of non-specific ST-T abnormalities and corrected QT-interval (QTc) duration in systemic lupus erythematosus (SLE) and rheumatoid arthritis (RA). (A) Mean prevalence and 95% CI of non-specific ST-T abnormalities in patients with SLE versus patients with RA in univariable analysis: 44% (32% to 56%) versus 17% (10% to 24%); OR=3.8 (1.8–7.7). *Adjusted for age and antimalarial use: 56% (40% to 71%) versus 17% (8% to 26%); OR=7.8 (2.4–25.8). (B) Mean QTc length and 95% CI in SLE versus RA in univariable analysis: 467 (455–478) versus 444 (438–449). **Adjusted for age, sex and aspirin use: 465 (451–480) versus 439 (432–446).

Forty-four per cent of the patients with SLE had non-specific ST segment and T-wave abnormalities (95% CI 32% to 56%) compared with 17% (10% to 24%) of the patients with RA (OR=3.8 (1.8 to 7.7); p=0.0001). After adjusting for age and antimalarial use, the identifiable confounders, this difference remained significant: 56% (40% to 71%) versus 17% (8% to 26%) in the SLE versus RA group, and the OR increased to 7.8 (2.4 to 25.8; p=0.0007) (figure 1A). In addition, the QTc duration was longer in patients with SLE compared with RA in both unadjusted and adjusted analyses (467 (455–478) vs 444 (438–449) ms, p=0.0001; and 465 (451–480) vs 439 (432–446) ms, p=0.002, respectively) (figure 1B). Restricting the analysis to only women (46 patients with SLE and 85 patients with RA, respectively), the mean QTc length was 468 (457–480) and 448 (442–455) in the SLE versus RA group; p=0.004.

Subsequently, we investigated the association between SLE and RA at different QTc duration cut-off points. Patients with SLE had a higher OR for having a QTc ≥420 ms, ≥440 ms, ≥460 ms and ≥480 ms compared with patients with RA (OR=3.2 (1–9.5; p=0.04); OR=3.5 (1.6–7.6; p=0.001); OR=3.0 (1.5–5.9; p=0.001) and 2.1 (0.9–4.6; p=0.06), respectively), meeting statistical significance in all but the QTc ≥480 ms category.

### Clinical and laboratory associations with non-specific ST-T abnormalities

Various characteristics were compared between patients with and without non-specific ST-T abnormalities in the SLE and RA groups (table 2). In patients with SLE, older age was associated with the presence of non-specific ST-T abnormalities; while in the RA group, men had a higher percentage of non-specific ST-T abnormalities. No association was seen between the prevalence of lupus nephritis and ECG abnormalities in patients with SLE. Similarly, anti-SSA/Ro and/or anti-SSB/La antibody positivity was not associated with ECG findings. In multivariable modelling of the covariates with a p value <0.25, no additional variables significantly associated with non-specific ST-T abnormalities were identified in the SLE or RA groups (p>0.05).

**Table 2:** Patient characteristics per strata of non-specific ST-T abnormalities

	Non-specific ST-T abnormalities in SLE (n=50)			Non-specific ST-T abnormalities in RA (n=139)		
	Present (n=22)	Absent (n=28)	p Value	Present (n=24)	Absent (n=115)	p Value
Age, years	41.0±15	32.8±9	**0.03**	61±8	59±8	0.29
Female sex, n (%)	20 (91%)	26 (93%)	0.80	10 (42%)	75 (65%)	**0.03**
Race/ethnicity						
White, n (%)	2 (9%)	0	0.19	20 (83%)	101 (88%)	0.52
Hispanic, n (%)	16 (73%)	21 (78%)	0.68	0	1 (1%)	0.65
Black, n (%)	4 (40%)	7 (60%)	0.73	0	1 (100%)	0.65
Disease duration, years	5 (3–10)	2.5 (0.5–8)	0.28	7 (4–14)	9 (4–16)	0.70
SLEDAI-2K	3 (0–10)	6 (2–12)	0.09	–	–	–
Lupus nephritis, n (%)	12 (54%)	10 (36%)	0.18	–	–	–
APS, n (%)	1 (4%)	2(7%)	1.0	–	–	–
DAS28	–	–	–	3.4±0.9	3.7±1	0.44
CRP, mg/L	10.5 (1.3–42.5)*	3.0 (0.6–24.8)†	0.22	2.2 (1.1–7.5)	2.1 (1–5.7)	0.93
IL-6, pg/mL	–	–	–	3.1 (1.8–7)	3.6 (1.5–7.7)	0.87
Antimalarials, n (%)	17 (77%)	12 (54%)	0.08	5 (21%)	18 (16%)	0.54
MMF, n (%)	11 (50%)	10 (36%)	0.31	–	–	–
AZA, n (%)	2 (9%)	3 (11%)	1.0	–	–	–
Non-biologic DMARD, n (%)	–	–	–	22 (92%)	95 (83%)	0.53
Biologic DMARD, n (%)	–	–	–	8 (33%)	58 (51%)	0.12
Glucocorticoids, n (%)	11 (50%)	16 (57%)	0.61	9 (37%)	41 (36%)	0.86
ds-DNA, antibody, n (%)	18 (82%)	23 (82%)	1.0	–	–	–
SSA/anti-Ro, n (%)	11 (50%)	17 (61%)	0.73	–	–	–
SSB/anti-La, n (%)	5 (23%)	11 (39%)	0.31	–	–	–
Sm antibody, n (%)	10 (21%)	14 (50%)	0.87	–	–	–
RNP antibody, n (%)	12 (54%)	17 (61%)	0.68	–	–	–
RF>40 units, n (%)	–	–	–	16 (67%)	69 (60%)	0.54
Anti-CCP>60 units, n (%)	–	–	–	14 (58%)	80 (70%)	0.26
Hypertension, n (%)	9 (41%)	9 (32%)	0.56	10 (42%)	46 (40%)	0.88
Diabetes, n (%)	2 (9%)	1 (4%)	0.57	2 (8%)	8 (7%)	0.68
Current smoking, n (%)	7 (32%)	5 (18%)	0.25	2 (8%)	11 (10%)	1.0
Aspirin, n (%)	3 (14%)	5 (18%)	1.0	6 (25%)	38 (33%)	0.44
Statin, n (%)	3 (14%)	2 (7%)	0.64	6 (25%)	17 (15%)	0.22
QT-modifying medication, n (%)	6 (27%)	10 (36%)	0.52	3 (12%)	21 (18%)	0.77
QTc, ms	465±48	469±34	0.73	452±37	442±32	0.21

Characteristics are expressed as n (%), as the mean±SD or as the median (IQR).

*Moderate–severe disease activity is defined as SLEDAI-2K >6 or DAS28 CRP >3.2. n=18.

†n=22.

Anti-CCP, anticitrullinated cyclic peptide; APS, antiphospholipid antibody syndrome; AZA, azathioprine; CRP, C reactive protein; DAS28, Disease Activity Score in 28 joints; DMARD, disease-modifying antirheumatic drug; IL-6, interleukin 6; MMF, mycophenolate mofetil; QTc, corrected QT-interval; RA, rheumatoid arthritis; RF, rheumatoid factor; SLE, systemic lupus erythematosus; SLEDAI-2K, Systemic Lupus Erythematosus Disease Activity Index 2000.

### Clinical and laboratory associations with QTc length

In patients with SLE, none of the assessed covariates were associated with QTc length when analysed continuously. Patients who used statins were less likely to have a QTc ≥420 ms (OR=0.1 (0–0.7; p=0.02) and ≥440 ms (OR=0.1 (0–0.9; p=0.04). In the RA group, female sex and high-sensitivity CRP level were associated with an increased length in QTc (parameter estimates of 12.2 (p=0.03) and 0.45 (p=0.046), respectively) (table 3). Furthermore, each unit higher DAS28-CRP score was associated with a 1.5-fold and 1.6-fold higher odds of having a QTc ≥460 ms and ≥480 ms, respectively (both p<0.05). Multivariable modelling (as above) did not reveal additional variables significantly associated with QTc length (p>0.05).

**Table 3:** Evaluation of the association of patient characteristics with QTc length

	SLE (n=50)		RA (n=139)	
	Parameter estimate	p Value	Parameter estimate	p Value
Age, years	0.37	0.41	0.38	0.25
Female sex	19.7	0.35	12.2	**0.03**
Race/ethnicity				
White	−39.5	0.17	−2.8	0.73
Hispanic	−13.5	0.32	14.2	0.67
Black	24.8	0.09	14.2	0.67
Disease duration, years	0.39	0.70	−0.32	0.31
SLEDAI-2K	−0.38	0.69	–	–
Lupus nephritis	−11.8	0.31	–	–
APS	−0.62	0.98	–	–
Moderate–severe disease activity*	−3.8	0.75	5.3	0.36
DAS28-CRP	–	–	3.2	0.24
CRP, mg/L	−0.03†	0.74	0.45	**0.046**
IL-6, pg/mL	–	–	0.68	0.23
Antimalarials	3.6	0.76	−0.15	0.98
MMF	−3.4	0.77	–	–
AZA	34.5	0.07	–	–
Non-biologic DMARD	–	–	−7.8	0.32
Biologic DMARD	–	–	−3.9	0.49
Glucocorticoids	−2.6	0.82	−0.7	0.90
ds-DNA antibody	12.8	0.39	–	–
SSA/anti-Ro	1.56	0.90	–	–
SSB/anti-La	−13.4	0.30	–	–
Sm antibody	2.6	0.83	–	–
RNP antibody	−1.49	0.90	–	–
RF>40 units	–	–	−0.50	0.93
Anti-CCP>60 units	–	–	0.80	0.89
Hypertension	−0.92	0.94	15.9	0.14
Diabetes	−3.1	0.90	3.1	0.59
Current smoking	−0.88	0.94	6.7	0.49
Aspirin	−10.0	0.52	−7.3	0.22
Statin	−25.9	0.17	8.8	0.2
QT-modifying medications	18.2	0.14	−4.9	0.51
Muscle relaxant, n (%)	−8.1	0.84	8.2	0.50
Antipsychotics, n (%)	−28.0	0.34	0	0
Antidepressants, n (%)	18.6	0.19	−7.6	0.31
Antimicrobials‡, n (%)	−35.6	0.39	9.8	0.68
Antiemetics, n (%)	−1.5	0.94	−8.0	0.68
ST-T abnormalities	−4.0	0.73	9.3	0.21

Univariable linear regression parameter estimates and corresponding p values of the association of QTc duration with each listed variable.

*Moderate–severe disease activity is defined as SLEDAI-2K >6 or DAS28 CRP >3.2.

†n=40.

‡Antimicrobials include macrolides, fluoroquinolones and HIV retrovirals.

Anti-CCP, anticitrullinated cyclic peptide; APS, antiphospholipid antibody syndrome; AZA, azathioprine; CRP, C reactive protein; DAS28, Disease Activity Score in 28 joints; DMARD, disease-modifying antirheumatic drug; IL-6, interleukin 6; MMF, mycophenolate mofetil; QTc, corrected QT-interval; RA, rheumatoid arthritis; RF, rheumatoid factor; SLE, systemic lupus erythematosus; SLEDAI-2K, Systemic Lupus Erythematosus Disease Activity Index 2000.

## Discussion

Our data show a higher prevalence of non-specific ST-T abnormalities and longer averaged QTc duration in patients with SLE compared with RA. Patients with SLE had an adjusted OR of 7.8 for having non-specific ST-T abnormalities relative to the patients with RA despite being younger, predominantly women and having shorter disease duration. The QTc duration in SLE was 26 ms longer compared with RA in the setting of a higher percentage of women, blacks, Hispanics and higher CRP levels in the SLE group. These ECG abnormalities were not associated with SLE-specific characteristics. To the best of our knowledge, this is the first study to evaluate non-specific ST-T and QTc abnormalities in a predominantly Hispanic and black cohort of patients with established SLE.

Excess death from CVD in patients with SLE and RA is well documented;^2^ ^10^ ^21–23^ traditional CV risk stratification scores greatly underperform in both diseases making it challenging to identify those at highest risk for CVD during the subclinical phase.^6^ The pathophysiology of cardiac involvement in SLE is multifactorial and in addition to accelerated atherosclerosis, vasculitis and myocarditis, every component of the heart can be involved in the inflammatory process and may contribute to the increased CV risk.^24^ As biopsies of cardiac tissue are invasive, costly and not routinely performed in clinical practice, a simple, inexpensive, practical and non-invasive diagnostic test such as the ECG could play an important role in CV risk stratification in SLE given its potential to detect early cardiac involvement.

Non-specific ST-T abnormalities can be found in 3–10% of ECGs of an otherwise healthy population,^25^ and these findings are known to predict an increased risk of CVD and mortality on long-term follow-up.^8^ ^26^ Although the study of cardiac conduction defects in SLE had been limited primarily to neonatal lupus in association with anti-SSA/Ro antibodies,^27^ a recent study that evaluated the prevalence of ECG abnormalities in an inception SLE adult cohort found that 31% of patients had non-specific ST-T abnormalities and 15% had a prolonged QTc;^7^ however, no comparison group was included and 74% of the patients were white and Asian. Our study addressed a gap in identifying the prevalence of these ECG abnormalities in a cohort of patients with SLE of mainly Hispanic and black origins, as well as at different clinical stages of the disease and hence with longer disease duration and a higher mean SLEDAI-2K score as compared with the patients studied by Bourré-Tessier *et al*.^7^ More importantly, the fact that in our study over half of the patients with SLE had non-specific ST-T abnormalities as compared with only 17% of the older, more male encompassing RA group is striking and underscores the need for evaluations that detect subclinical cardiovascular involvement in SLE. Although the clinical impact of non-specific ST-T ECG abnormalities in patients with SLE without CVD is unknown, it is enticing to speculate that they represent subclinical CVD. However, studies linking ECG abnormalities to clinical CV outcomes in SLE are lacking.

In agreement with our findings, the association between inflammatory marker levels, such as CRP and IL-6, with ECG abnormalities has been previously described in patients with RA.^28–31^ Other studies have suggested a CV protective effect for medications such as hydroxychloroquine, methotrexate and antitumour necrosis factor inhibitors;^32–34^ however, we did not find these associations in our study. Interestingly, the use of statins among patients with SLE seemed to confer less risk of having a longer QTc duration.

The length of the QTc is an independent cardiovascular risk factor.^35^ ^36^ Both moderate (QTc of 420–440 ms) and extensive QTc prolongation (>440 ms) are predictive of all-cause mortality in healthy middle-aged populations.^37^ In patients with RA, Panoulas *et al*^11^ showed that a 50 ms increase in the QTc interval was associated with a doubling of the risk of all-cause mortality, an association mediated by CRP levels. While sudden cardiac death is now recognised as an important cause of death in patients with SLE,^38^ the pathophysiological mechanisms that lead to it remain unclear. Lazzerini *et al*^39^ reported an association between QTc prolongation and the development of complex ventricular arrhythmias in patients with different connective tissue diseases and anti-SSA/Ro antibodies. In their follow-up work, this group reported that in patients with connective tissue diseases, QTc prolongation correlated with only one of the subtypes of the anti-SSA/Ro antibodies, specifically those recognising the 52-kd subunit.^29^ In our study, the longer QTc in the patients with SLE showed no association with anti-SSA/Ro antibodies, and similar to our findings, other investigators have not been able to replicate this association.^7^ ^40^ However, it is possible that the above-mentioned lack of association is due to low circulating levels of the specific anti-SSA/Ro 52-kd antibody subtype among the studied patients. Indeed, in an unselected group of patients presenting with Torsades de Pointes, the main autoantibody subtype present was the anti-SSA/Ro 52-kd subtype.^41^ This correlation is attributed to the homology between the anti-SSA/Ro-52-kd antigen protein and a subunit of the rapidly activating delayed potassium channel; the cross-interaction thus impairs ventricular cardiac repolarisation.^42^

We acknowledge certain limitations for this study. While we focused on ECG as a marker of cardiovascular health, the ECG lacks specificity to detect early/preclinical myocardial lesions. However, non-specific ST-T abnormalities and prolongation of the QTc interval are known to correlate with an increased future risk of CVD.^8^ ^26^ ^43^ Also, our study is limited by the lack of a healthy control group. Nonetheless, it has been reported that in healthy 25-year-old to 44-year-old women, the prevalence of non-specific ST-T ECG abnormalities is 3–4%, whereas in Hispanic communities these ECG findings are found in 5–7% of the population.^25^ ^44^ We used patients with RA as a comparison group given their known excess cardiovascular risk and mortality. A limitation of doing so was the difference in race/ethnicity between the SLE and the RA groups, with Hispanics/blacks being the predominant ethnicity in the former and whites in the latter. In patients with known heart disease, the findings of conduction abnormalities and longer QT intervals are more prevalent among Hispanics and blacks as compared with whites.^45^ However, in the large Multi-Ethnic Study of Atherosclerosis (MESA) in the general population without clinical CVD, out of more than 6000 patients studied, over 40% were Hispanics and blacks, and no interaction between ethnicity and QTc prolongation was found.^46^ Similarly, in the same MESA cohort, the prevalence of non-specific ST-T abnormalities was 14% with no increased risk in Hispanics.^47^ Yet another limitation of using the patients with RA as a control group is the challenge of comparing disease activity between the two groups. It is known that QTc length and QT dispersion correlate with disease activity and cytokine levels in both patients with SLE and patients with RA.^7^ ^11^ ^30^ ^48–51^ We used the SLEDAI-2K, DAS28-CRP and CRP levels (as well as IL-6 levels in the RA group) to estimate disease activity. CRP levels were lower in the RA cohort as compared with the SLE cohort, and the IL-6 levels in the RA group were approximately three times lower than what has been reported in patients with active disease.^52^ IL-6 levels were not available for comparison in the SLE group. It is therefore possible that differences in the degree of systemic inflammation between the two groups limit the extrapolation of our findings. However, the mean DAS28-CRP in the studied patients with RA was 3.6 and the median SLEDAI-2K in the SLE group was 6, suggesting at least moderate disease activity in both groups by these standardised validated scores. In addition, we did not test for anti-SSA antibody subtypes, which could have prevented finding an association between ECG abnormalities and these autoantibodies.

Finally, although the sample size was small for the SLE group, we nonetheless observed statistically significant differences in the prevalence of ECG abnormalities between the patients with SLE and patients with RA suggesting a robust finding.

In conclusion, patients with SLE had a higher prevalence of non-specific ST-T abnormalities and longer QTc on ECG compared with patients with RA. Further longitudinal studies are needed to evaluate the progression of these ECG findings and define the long-term impact on cardiovascular morbidity and mortality.
